# Cyclin Y phosphorylation- and 14-3-3-binding-dependent activation of PCTAIRE-1/CDK16

**DOI:** 10.1042/BJ20150486

**Published:** 2015-07-23

**Authors:** Saifeldin N. Shehata, Maria Deak, Nicholas A. Morrice, Eriko Ohta, Roger W. Hunter, Vera M. Kalscheuer, Kei Sakamoto

**Affiliations:** * Nestlé Institute of Health Sciences SA, EPFL Innovation Park, bâtiment G, 1015 Lausanne, Switzerland; † School of Life Sciences, Ecole Polytechnique Fédérale de Lausanne (EPFL), 1015 Lausanne, Switzerland; ‡ The Beatson Institute for Cancer Research, Garscube Estate, Switchback Road, Bearsden, Glasgow G61 1BD, Scotland, U.K.; § MRC Protein Phosphorylation and Ubiquitylation Unit, College of Life Sciences, University of Dundee, Dow Street, Dundee DD1 5EH, Scotland, U.K.; ∥ Max Planck Institute for Molecular Genetics, Department of Human Molecular Genetics, Ihnestr. 73, D-14195 Berlin, Germany

**Keywords:** 14-3-3, cyclin-dependent kinase (CDK), intellectual disability, mass spectrometry, protein kinase

## Abstract

PCTAIRE-1 [also known as cyclin-dependent kinase 16 (CDK16)] is implicated in various physiological processes such as neurite outgrowth and vesicle trafficking; however, its molecular regulation and downstream targets are largely unknown. Cyclin Y has recently been identified as a key interacting/activating cyclin for PCTAIRE-1; however, the molecular mechanism by which it activates PCTAIRE-1 is undefined. In the present study, we initially performed protein sequence analysis and identified two candidate phosphorylation sites (Ser^12^ and Ser^336^) on cyclin Y that might be catalysed by PCTAIRE-1. Although *in vitro* peptide analysis favoured Ser^12^ as the candidate phosphorylation site, immunoblot analysis of cell lysates that had been transfected with wild-type (WT) or kinase-inactive (KI) PCTAIRE-1 together with WT or phospho-deficient mutants of cyclin Y suggested Ser^336^, but not Ser^12^, as a PCTAIRE-1-dependent phosphorylation site. Monitoring phosphorylation of Ser^336^ may provide a useful read-out to assess cellular activity of PCTAIRE-1 *in vivo*; however, a phospho-deficient S336A mutant displayed normal interaction with PCTAIRE-1. Unbiased mass spectrometry and targeted mutagenesis analysis of cyclin Y identified key phosphorylation sites (Ser^100^ and Ser^326^) required for 14-3-3 binding. Recombinant WT cyclin Y, but not a S100A/S326A mutant, prepared in COS-1 cells co-purified with 14-3-3 and was able to activate bacterially expressed recombinant PCTAIRE-1 in cell-free assays. Finally, we observed that recently identified PCTAIRE-1 variants found in patients with intellectual disability were unable to interact with cyclin Y, and were inactive enzymes. Collectively, the present work has revealed a new mechanistic insight into activation of PCTAIRE-1, which is mediated through interaction with the phosphorylated form of cyclin Y in complex with 14-3-3.

## Abstract (precis)

We have identified a key molecular mechanism by which cyclin Y activates atypical cyclin-dependent kinase 16 (CDK16)/PCTAIRE-1, which involves 14-3-3 binding to cyclin Y through phosphorylation of two residues, namely Ser^100^ and Ser^326^.

## INTRODUCTION

The human cyclin-dependent kinase 16 (*CDK16*) gene, which encodes the protein kinase PCTAIRE-1 (also known as CDK16), maps to the X chromosome (Xp11.3) whose defects and copy-number variants have been linked to various diseases, including intellectual disability and related disorders [1]. As a member of the CDK family, early studies focused on the role of PCTAIRE-1 in cell-cycle regulation. Although still controversial, there is no established evidence that PCTAIRE-1 plays a critical role in cell-cycle control [2–4]. PCTAIRE-1 is expressed in post-mitotic cells and displays a wide tissue distribution with highest abundance in brain and testis [5,6] and has been implicated in diverse biological and physiological processes, including neurite outgrowth [3,7], vesicle trafficking [8,9], spermatogenesis [5], glucose homoeostasis [10,11] and, most recently, muscle differentiation [12].

PCTAIRE-1 is one of three members of the PCTAIRE family (namely PCTAIRE-1, -2 and -3). PCTAIRE is composed of three principal domains: a central kinase domain that is highly conserved between the three isoforms (~80% protein sequence identity), flanked by a long N-terminus and a short C-terminus of which the extreme N-terminus is unique to each isoform [2,13]. Until recently, PCTAIRE and its closely related kinase PFTAIRE (PFTAIRE-1/2, also known as CDK14/15) were considered ‘orphan CDKs’, because their putative cyclin partners were unknown and thus termed according to a characteristic α-helical sequence that corresponds to the ‘PSTAIRE’ helix in the cyclin-binding domain of CDK1 or CDK2 [13]. However, recent studies identified a key cyclin-binding partner, namely cyclin Y. The original work implicating that cyclin Y might be a potential partner for PCTAIRE-1 came from a large-scale yeast two-hybrid screen in *Drosophila melanogaster*, where Eip63E (ecdysone-induced protein encoded by a gene at chromosomal position 63E), most similar to the mammalian PFTAIRE-1/CDK14, interacted with a novel cyclin-like protein, coded as CG14939. Subsequent studies confirmed interaction between Eip63E and CG14939 (renamed ‘cyclin Y’) in *Drosophila* cells [14] and between human PFTAIRE-1 and cyclin Y [15]. Ou et al. [16] have demonstrated that the *Caenorhabditis elegans* homologue of PCTAIRE-1, PCT1, bound to CYY1, the *C. elegans* homologue of cyclin Y. Subsequently, Mikolcevic et al. [5] reported that mammalian PCTAIRE-1 also interacts with cyclin Y, and we have demonstrated that binding of PCTAIRE-1 to cyclin Y increases (>100-fold) PCTAIRE-1 catalytic activity [6].

Although PCTAIRE-1 is implicated in a wide variety of cellular and physiological processes, the molecular basis of its regulation as well as downstream pathway(s) leading to such physiological outcomes remains elusive. Since no *bona fide* substrates for PCTAIRE-1 have been identified and the phosphorylation consensus sequence had not been defined, we recently performed positional scanning using a peptide library. We identified that PCTAIRE-1 has a unique substrate preference compared with other CDK members and developed a novel peptide substrate termed ‘PCTAIRE-tide’ [6]. This enabled us to more robustly and quantitatively measure PCTAIRE-1 activity to study its molecular regulation.

In the present study, we initially undertook protein sequence analysis of human cyclin Y and identified two conserved proline-directed serine residues (Ser^12^ and Ser^336^) that resemble the preferred consensus sequence for PCTAIRE-1. This led us to hypothesize that PCTAIRE-1 phosphorylates cyclin Y and that this phosphorylation may at least partly influence their interaction and hence activity. We have identified Ser^336^ as a PCTAIRE-1-dependent phosphorylation site, but found that the phosphorylation of Ser^336^ has no significant impact on interaction between PCTAIRE-1 and cyclin Y. Interestingly, however, analysis of additional phosphorylation sites on cyclin Y revealed that phosphorylation of Ser^100^ and Ser^326^ plays an important role in binding and activating PCTAIRE-1 by promoting binding to 14-3-3 proteins. We also report that recently identified PCTAIRE-1 variants found in patients with intellectual disability were unable to interact with cyclin Y and are inactive enzymes.

## EXPERIMENTAL

### Materials

Peptide substrates for kinase assays were synthesized by GL Biochem. [γ-^32^P]ATP was from PerkinElmer. Horseradish peroxidase (HRP)-conjugated secondary antibodies were from Jackson Immuno Research. P81 paper was from Whatman. All cell culture reagents were from Life Technologies. Unless otherwise indicated all other reagents were from Sigma.

### Antibodies

Anti-PCTAIRE-1 (C-16), anti-PCTAIRE-1 (G6.1) and anti-14-3-3 (K-19) antibodies were from Santa Cruz Biotechnology. Anti-cyclin Y antibody was from Proteintech. Anti-haemagglutinin (HA) antibody was from Covance Research Products. The following antibodies were raised in rabbit by YenZym Antibodies against the indicated immunogens where denotes a phosphoamino acid: pSer^12^-cyclin Y (YZ4631, VSSS*PKLRRNAHC-NH_2_), pSer^100^-cyclin Y (YZ4891 second cycle, QIARKYSS*CSTIFLD-NH_2_), pSer^326^-cyclin Y (YZ3909, RKRSAS*ADNLTLPC-NH_2_) and pSer^336^-cyclin Y (YZ4633 second cycle, CDNLTLPRWS*PAIIS-NH_2_). The following antibodies were raised in sheep by the Division of Signal Transduction Therapy (University of Dundee) against the indicated immunogens: GST (S902A, third bleed, GST from *Schistosoma japonicum*), cyclin Y (S206D, third bleed, full-length human GST–cyclin Y).

### Cloning, mutagenesis and sequence analysis

All plasmid constructs were generated using standard molecular biology techniques. The coding regions of PCTAIRE-1 isoform 1 (NM_006201.4) and 14-3-3ζ (NM_003406.3) were amplified from human placental RNA, cyclin Y (NM_145012.4) from testes RNA (Agilent Technologies) using Superscript III One Step RT-PCR kit (Life Technologies). The resulting PCR products were subcloned into an intermediate vector pJET (Fermentas) or directly into the mammalian and bacterial expression vectors, pCMV5 (AF239249.1) and pGEX-6P-1 (GE Healthcare). Site-directed mutagenesis was performed using the QuikChange method (Agilent) using KOD Hot Start DNA Polymerase (Novagen). The sequence of all constructs was verified utilizing the BigDyeR Terminator 3.1 kit and a 3500XL Genetic analyser (Life Technologies) in-house at the Nestlé Institute of Health Sciences. Sequence alignment was performed using the Muscle algorithm, edited using the Jalview alignment editor [17] and displayed using BOXSHADE (http://www.ch.embnet.org/software/BOX_form.html).

### Cell culture, transfection and harvesting

COS-1 cells (A.T.C.C.) were maintained in high-glucose Dulbecco's modified Eagle's medium (DMEM) supplemented with 10% (v/v) FBS under 5% CO_2_. Cells were transfected with DNA using polyethyleneimine and harvested 36 h post-transfection. Cells were washed with ice-cold PBS and scraped into lysis buffer (50 mM Tris/HCl, pH 7.5, 1 mM EDTA, 1 mM EGTA, 0.27 M sucrose, 1% (w/v) Triton X-100, 50 mM NaF, 5 mM Na_4_P_2_O_7_, 1 mM Na_3_VO_4_, 1 mM DTT, 1 mM benzamidine and 0.5 mM PMSF). Lysates were clarified at 17000 ***g*** for 10 min at 4°C and stored at −80°C. Protein concentration was determined using Bradford reagent and BSA standard.

### Mass spectrometry (MS) analysis of HA–cyclin Y

COS-1 cells were transfected with HA-tagged human cyclin Y with or without co-transfection with FLAG–PCTAIRE-1 and lysates were prepared 36 h later as described above. HA–cyclin Y was immunoprecipitated from 2 mg of lysate using HA–agarose, washed twice with 0.5 ml of lysis buffer plus 0.5 M NaCl, twice with 0.5 ml of buffer A (50 mM Tris/HCl, pH 8, 0.1 mM EGTA and 1 mM DTT) and eluted with Laemmli sample buffer. Eluted HA–cyclin Y was separated by SDS/PAGE and stained with colloidal Coomassie (Blue Life Technologies). The HA–cyclin Y band was excised, destained, in-gel reduced and alkylated with iodoacetamide (50 mM), then dried with acetonitrile followed by Speed Vac concentration. Samples were digested with 60 μl of 2 μg/ml trypsin (sequencing grade, Promega) in 50 mM triethylammonium bicarbonate (TEAB), pH 8, overnight. Peptides were extracted with an equal volume of acetonitrile, dried and redissolved in 60 μl of 5% acetonitrile/10 mM TEAB. Samples were analysed by LC–MS on a 180 mm×0.075 mm packed emitter (Reprosil C_18_ 3 μM) coupled to an Orbitrap Velos MS system. After trapping the peptides on a 20 mm×0.1 mm C_18_ trap column (Nanoseparations) equilibrated in buffer A (2% acetonitrile/0.1% formic acid in water) at 7 ml/min, peptides were separated using a discontinuous gradient from 98% buffer A:2% buffer B (80% acetonitrile/20% water/0.1% formic acid) to 80% buffer B over 60 min at 300nl/min. The ten most abundant precursors detected in the Orbitrap at 30000 resolution (2–4^+^ charge state) were selected for MS/MS in the LTQ Velos. To ensure the best fragmentation spectrum for any phosphopeptide was obtained, the precursors were fragmented using multistage activation (MSA) of the precursor mass (*m/z*) minus 49, 32.33 and 24.25 Da (this represents the loss of H_3_PO_4_ from a 2^+^, 3^+^ or 4^+^ precursor ion). Raw files were processed using Proteome Discoverer 1.4 and searched using Mascot 2.3 against the Swiss Prot database (human only), allowing for carbamidomethyl modification of cysteine, oxidation of methionine and phosphorylation of serine/threonine/tyrosine.

### Preparation of GST-tagged proteins in *Escherichia coli*

GST-tagged human PCTAIRE-1, human cyclin Y and human 14-3-3ζ in pGEX-6P were expressed in *Escherichia coli* BL21-CodonPlus(DE3)RIL cells and isolated by column purification using glutathione–Sepharose 4B. Briefly, transformed *E. coli* cells were cultured in LB medium until a OD_600_ of 0.6 was attained and induced with 0.2 mM IPTG for 16 h at 18°C. Cells were pelleted by centrifugation at 5000 ***g*** for 30 min, lysed in 50 mM Tris/HCl, pH 7.5, 0.25 M NaCl, 0.1 mM EDTA, 1 mM benzamidine/HCl and 1 mM DTT by sonication and clarified by centrifugation at 30000 ***g*** for 30 min. Supernatants were incubated with glutathione–Sepharose 4B for 1 h at 4°C, washed extensively with lysis buffer and eluted with 20 mM reduced glutathione in 50 mM Tris/HCl, pH 8. Preparations were gel filtered over Sephadex G-25 to remove excess glutathione and the GST-tag was either retained or cleaved using human rhinovirus (HRV) 3C protease. Protein concentration was estimated by densitometry of Coomassie Blue-stained gels using BSA standards. Protein preparations were snap-frozen in liquid nitrogen and stored at −80°C in 50 mM Tris/HCl, pH7.5, 0.15 M NaCl, 0.1 mM EDTA and 10% (v/v) glycerol.

### Baculovirus protein expression and purification

N-terminal hexahistidine (His_6_)-tagged cyclin Y wild-type (WT) and S326A constructs were used to generate recombinant baculovirus using the Bac-to-Bac system (Life Technologies) following the manufacturer's protocol. Baculovirus protein expression and purification was performed by the DSTT. *Spodoptera frugiperda* Sf 21 cells (1.5×10^6^/ml) were infected at a multiplicity of infection of 5 and harvested 48 h post-infection. His_6_-tagged proteins were affinity purified using Ni-NTA (Ni2^+^-nitrilotriacetic acid)–agarose.

### Preparation of tissue lysates

Animal studies were approved by the local Ethical Committee of the Canton of Vaud, Switzerland, and performed under licence number 2519. C57BL/6N mice were obtained from Charles River Laboratories. Mice were killed by CO_2_ exposure and tissues were rapidly dissected and frozen in liquid nitrogen. Tissues were homogenized using a rotor-stator homogenizer (Polytron, Kinematica AG) in lysis buffer, clarified at 17000 ***g*** for 10 min at 4°C and stored at −80°C. Protein concentration was determined using Bradford reagent and BSA as standard.

### Immunoprecipitation

Tissue lysates were incubated with 1 μg of anti-cyclin Y S206D antibody and 5 μl of Protein G–Sepharose for 1 h at 4°C. FLAG- and HA-tagged proteins were isolated from cells using 5 μl of FLAG M2–or HA–agarose respectively. Immune complexes were pelleted at 500 ***g*** for 1 min and washed twice with 0.1 ml of lysis buffer plus 0.5 M NaCl, twice with 0.1 ml of buffer A and eluted with Laemmli sample buffer for analysis by immunoblotting or assayed directly for kinase activity.

### Immunoblotting

Cell/tissue lysates were denatured in Laemmli buffer, separated by Tris/glycine SDS/PAGE and transferred onto PVDF a membrane. Membranes were blocked for 1 h at room temperature in 20 mM Tris/HCl (pH 7.6), 137 mM NaCl, 0.1% (v/v) Tween 20 (Tris buffered saline with Tween-20; TBST) containing 5% (w/v) skimmed milk. Membranes were incubated in primary antibody prepared in TBST containing 5% (w/v) BSA overnight at 4°C. Signal detection was performed using HRP-conjugated secondary antibodies and enhanced chemiluminescent reagent.

### Kinase assay

Purified recombinant PCTAIRE-1 or immune complexes isolated from cell/tissue lysates were assayed for phosphotransferase activity in a final assay volume of 50 μl containing 50 mM HEPES, pH 7.5, 0.1 mM EGTA, 10 mM magnesium acetate, 0.1 mM [γ-^32^P]ATP (300 c.p.m./pmol), 1 mM DTT and 50 μM PCTAIRE-tide (PKSPKARKKL) or the indicated concentrations of peptide substrate. Reactions were incubated at 30°C and terminated by spotting onto P81 paper and immersion in 75 mM H_3_PO_4_. P81 filters were washed three times for 10 min with H_3_PO_4_, rinsed with acetone, air-dried and incorporation of γ-^32^P determined by Cherenkov counting. Results are expressed in units·mg^−1^, where 1 unit is defined as the incorporation of 1 nmol of phosphate·min^−1^.

### 14-3-3 binding/competition assay

FLAG–cyclin Y was immunoprecipitated from 50 μg of COS-1 lysates and washed as described above, then incubated for 20 min at room temperature with 1 mM 14-3-3-binding phosphopeptide (ARAAS*APA peptide, where S* denotes phosphoserine) in TBS (50 mM Tris, 150 mM NaCl, 1 mM benzamidine/HCl, 1 mM DTT and 0.03% Briji35). Non-phosphopeptide (ARAASAPA) was used as negative control. After washing with ice-cold TBS, 0.1 μg of recombinant PCTAIRE-1 and/or 0.5 μg 14-3-3ζ (both from *E. coli*) were added together or in isolation and incubated for a further 20 min in TBS at room temperature. The immune complexes were then washed and eluted with Laemmli sample buffer for analysis by immunoblotting.

## RESULTS

### Sequence and peptide analysis identified potential PCTAIRE-1 phosphorylation sites on cyclin Y

**Figure 1:**
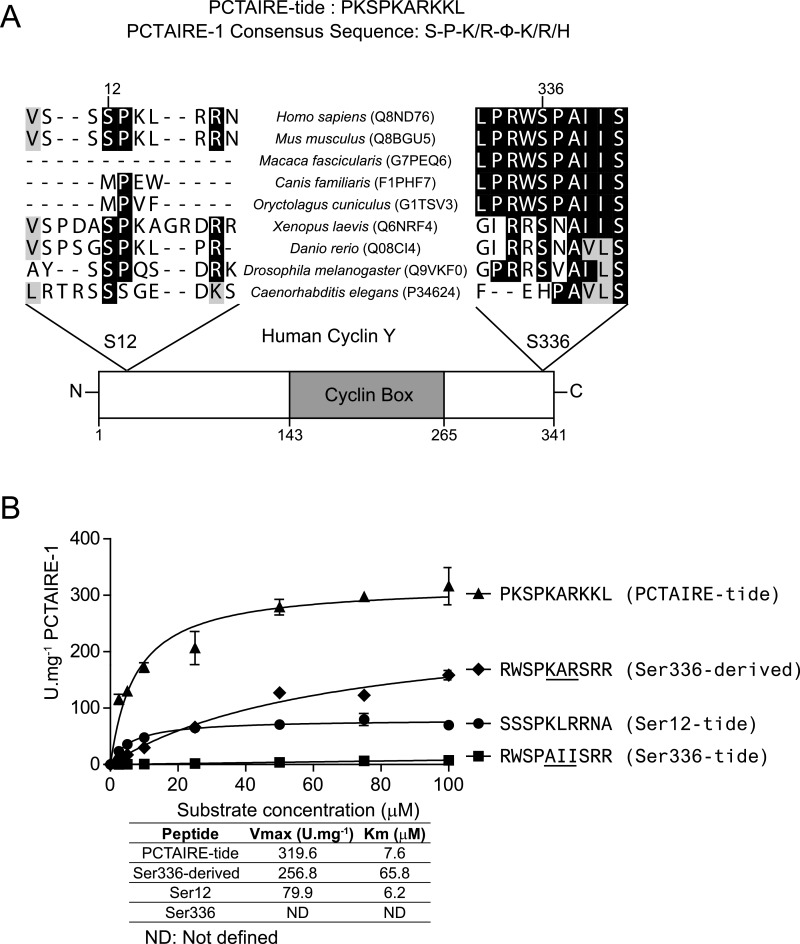
Analysis of S-P motifs on human cyclin Y (**A**) Diagram illustrating the domain organization of human cyclin Y with a global multiple sequence alignment of amino acid sequences surrounding the S-P motifs. Uni Prot accession numbers are indicated in parentheses. (**B**) The *in vitro* phosphotransferase activity of FLAG–PCTAIRE-1–HA–cyclin Y complex was determined using peptides derived from residues surrounding Ser^12^ and Ser^336^ on human cyclin Y, as well as a derivative of the Ser^336^ peptide based on the PCTAIRE-tide sequence (changes underlined). Peptide concentration was varied over the range 0–100 μM and substrate saturation curves were fitted using non-linear regression to the Michaelis–Menten model. Fitted parameters [*K*_m_ (μM) and *V*_max_ (units·mg^−1^)] are listed in the adjoining table. Results are expressed as means±S.D. and are representative of three independent experiments.

Our recent positional scanning peptide library analysis has revealed key substrate-specificity requirements of PCTAIRE-1 [6]. We found that although an absolute requirement for a proline residue immediately C-terminal to the phospho-acceptor site (+1) and preference for a basic residue at +2 are similar to other conventional CDKs, some elements (preference for a basic residue at +4, but not at +3) were unique to PCTAIRE-1. We established a preferred consensus sequence of S-P-K/R-ϕ-K/R/H (ϕ, represents a small aliphatic amino acid) and generated an optimal substrate peptide (PCTAIRE-tide, PKSPKARKKL) for robust measurements of recombinant and endogenous PCTAIRE-1 kinase activity *in vitro* [6]. Analysis of the protein sequence of human cyclin Y, a cognate binding partner of PCTAIRE-1 necessary for its activation [5,6], identified two proline-directed serine residues (S-P motif; Figure 1A). One of the S-P motifs (containing Ser^336^ in the human sequence) is well conserved among mammals but not in lower organisms such as *D. melanogaster* and *C. elegans*, whereas the other S-P motif (containing Ser^12^) is conserved in humans and mouse, as well as the majority of the lower organisms inspected. We noted that residues surrounding Ser^12^ (**S**PKLRRN) of human cyclin Y resemble the preferred consensus sequence for PCTAIRE-1 (Figure 1A). Thus we hypothesized that PCTAIRE-1 phosphorylates cyclin Y and that this modification influences their interaction and hence catalytic activity of PCTAIRE-1. To test this hypothesis, we initially generated synthetic peptides encompassing residues surrounding Ser^12^ (termed Ser^12^-tide) and Ser^336^ (plus an additional two arginine residues to enable efficient binding of the peptide to P81 paper; termed Ser^336^-tide) of human cyclin Y and determined *in vitro* substrate kinetics using recombinant PCTAIRE-1–cyclin Y complex isolated from COS-1 cells. Ser^12^-tide demonstrated a comparable *K*_m_ to that of PCTAIRE-tide (6.2 μM compared with 7.6 μM), but displayed an approximately 4-fold lower *V*_max_ (Figure 1B). In contrast, no detectable activity above background was observed for Ser^336^-tide, possibly due to absence of a preferred basic residue at +2. In support of this, alteration of +2 (alanine) and +4 (isoleucine) amino acids to lysine and arginine respectively in Ser^336^-tide to more resemble PCTAIRE-tide robustly increased PCTAIRE-1 activity. However, the *K*_m_ of the modified Ser^336^-tide was still 10-fold higher than that of Ser^12^- or PCTAIRE-tide (Figure 1B). Taken together, *in vitro* peptide analysis suggests that Ser^12^, but not Ser^336^, is potentially a candidate for PCTAIRE-1-targeted phosphorylation.

### Detection and validation of PCTAIRE-1-dependent phosphorylation of cyclin Y

**Figure 2:**
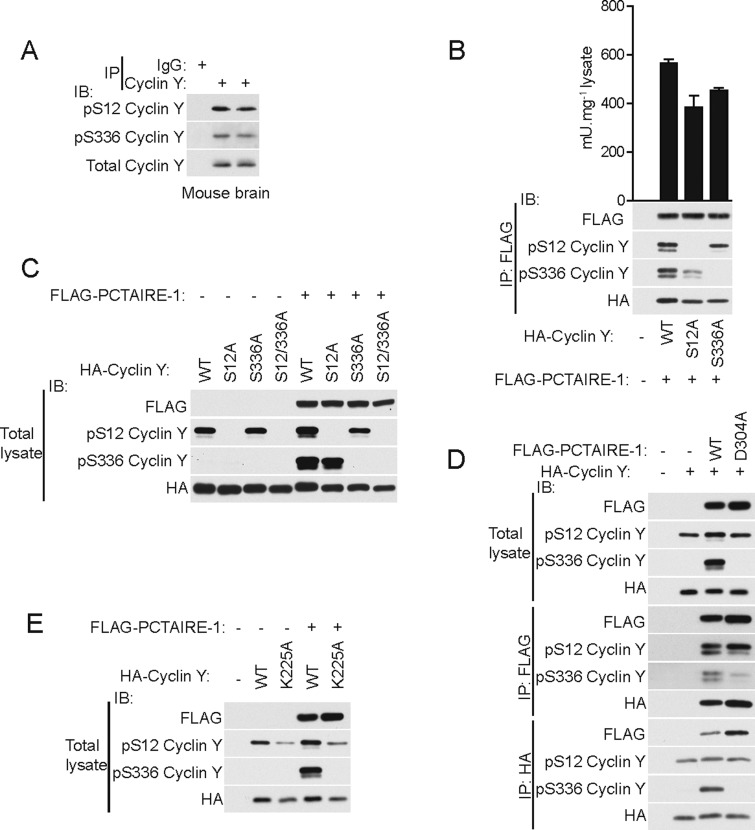
Ser^336^ cyclin Y phosphorylation is PCTAIRE-1-dependent (**A**) Endogenous cyclin Y was immunoprecipitated (IP) from 0.1 mg of brain homogenates of two C57BL/6N mice using anti-cyclin Y antibody and immunoblotted (IB) using the indicated antibodies. (**B**) WT or serine-to-alanine mutants of the HA–cyclin Y S-P motifs were co-transfected with FLAG–PCTAIRE-1 in COS-1 cells. Lysates were immunoprecipitated using FLAG–agarose and either immunoblotted with the indicated antibodies or assayed for PCTAIRE-1 kinase activity. (**C**) WT or serine-to-alanine (single or double) mutants of the HA–cyclin Y S-P motifs were transfected in COS-1 cells with or without FLAG–PCTAIRE-1 and total lysates immunoblotted using the indicated antibodies. (**D**) HA–WT cyclin Y was expressed in COS-1 cells with or without FLAG WT PCTAIRE-1 or kinase inactive (D304A) mutant and total lysates immunoblotted with the indicated antibodies. Alternatively, lysates were immunoprecipitated using FLAG–or HA–agarose followed by immunoblotting. (**E**) HA–WT cyclin Y or K225A (binding-deficient mutant) were transfected in COS-1 cells with or without FLAG–WT PCTAIRE-1 or KI (D304A) mutant and total lysates were immunoblotted using the indicated antibodies. Results are expressed as means±S.D. and are representative of three independent experiments.

To validate *in vitro* results obtained using short synthetic peptides (Figure 1) and further explore whether the two potential PCTAIRE-1 phosphorylation sites on full-length cyclin Y (Ser^12^ and Ser^336^) are phosphorylated in intact cells/tissues, we first immunoprecipitated endogenous cyclin Y from mouse brain extracts and immunoblotted with phospho-specific cyclin Y antibodies. As illustrated in Figure 2(A), we could detect phosphorylation of both Ser^12^ and Ser^336^ in these extracts. To examine whether phosphorylation of either site influences the cyclin Y interaction with/activation of PCTAIRE-1, we ectopically co-expressed FLAG–WT PCTAIRE-1 and HA–WT cyclin Y or phospho-deficient alanine mutants (S12A or S336A) in COS-1 cells. We detected phosphorylation of both Ser^12^ and Ser^336^ in WT cyclin Y bound to PCTAIRE-1 and observed that the single phospho-deficient mutants could still interact with and activate PCTAIRE-1 (Figure 2B). A modest reduction in PCTAIRE-1 activity in the alanine mutants compared with WT was observed, which could be due to slightly lower expression of the mutants (Figure 2B). It can also be noted that the double S12A/S336A mutant bound and activated PCTAIRE-1 similarly to the WT and single mutants (results not shown). Interestingly, but contrary to the results obtained using synthetic peptides in the cell-free assay (Figure 1B), we found that whereas Ser^12^ phosphorylation can be detected irrespective of the presence of PCTAIRE-1, Ser^336^ phosphorylation could only be observed when PCTAIRE-1 was co-expressed (Figure 2C). We confirmed that PCTAIRE-1-dependent phosphorylation of Ser^336^ requires intact catalytic activity, as a catalytically inactive (D304A) mutant of PCTAIRE-1 (which retains the ability to bind cyclin Y) abolished Ser^336^ phosphorylation (Figure 2D). This is consistent with a recent study reporting that PCTAIRE-1 phosphorylates Ser^336^ on cyclin Y in cell-free assays, although the result was not shown [5]. To further demonstrate that interaction of PCTAIRE-1 with cyclin Y is critical for Ser^336^ phosphorylation, we overexpressed a cyclin Y mutant (K225A) that cannot bind to PCTAIRE-1 [6]. Although Ser^12^ phosphorylation was detectable in the mutant cyclin Y (albeit to a lesser degree compared with WT cyclin Y, presumably due to lower expression), Ser^336^ could be seen in WT cyclin Y when co-expressed with PCTAIRE-1, but not in the K225A mutant (Figure 2E). Taken together, whereas *in vitro* peptide analysis favoured Ser^12^ over Ser^336^ as PCTAIRE-1 targeted phosphorylation site (Figure 1B), cellular experiments using full-length recombinant cyclin Y have revealed Ser^336^ as a PCTAIRE-1-dependent phosphorylation site.

### Phospho-peptide mapping identified multiple phosphorylation sites on cyclin Y

**Figure 3:**
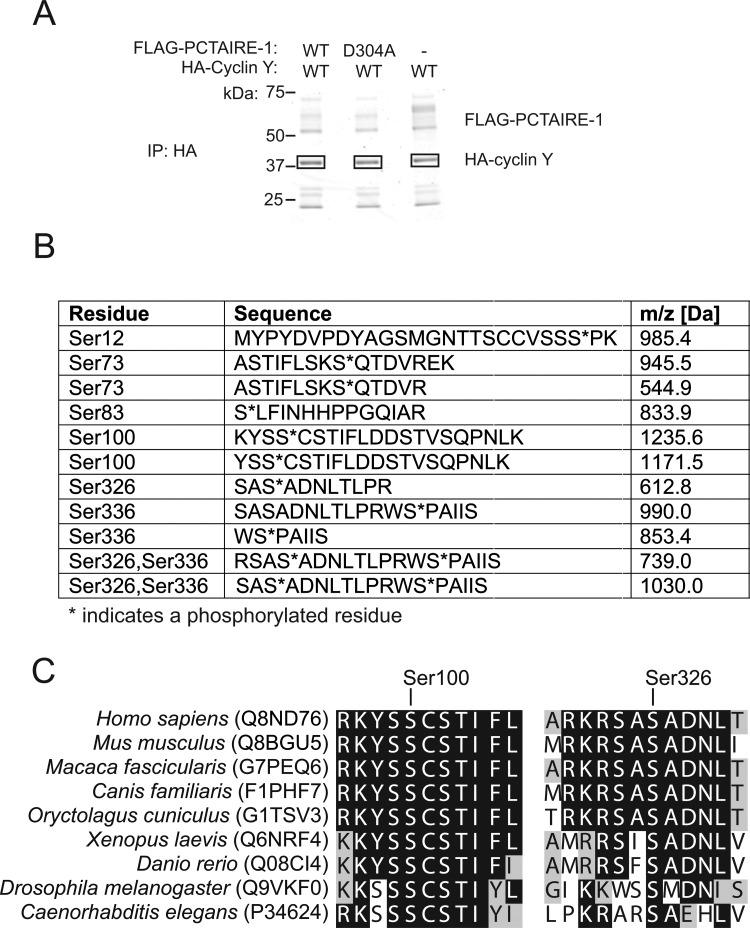
Mass spectrometry (MS)-based mapping of phospho-sites on human cyclin Y (**A**) HA–cyclin Y expressed in COS-1 cells with or without FLAG–WT PCTAIRE-1 or KI (D304A) mutant was immunoprecipitated (IP) using HA–agarose and separated by SDS/PAGE. The gel was stained with colloidal Coomassie. Rectangular boxes indicate where gel pieces were excised and analysed by MS. (**B**) Sequences and *m*/*z* scores of peptides identified during MS analysis of samples excised in (**A**) that correspond to phosphorylation sites on human cyclin Y. (**C**) Global multiple sequence alignment of human cyclin Y with the indicated orthologues showing amino acid sequences surrounding Ser^100^ and Ser^326^. MS experiments were repeated three times.

To determine whether there were other phosphorylation sites on cyclin Y that are important for interaction with/activation of PCTAIRE-1, we performed phosphopeptide mapping of cyclin Y. Human HA–WT cyclin Y was transfected alone or with human FLAG–WT PCTAIRE-1 or kinase-inactive (KI) FLAG–PCTAIRE-1 (D304A) in COS-1 cells. HA–cyclin Y proteins were affinity-purified, separated by SDS/PAGE (Figure 3A) and in-gel digested with trypsin. The resulting peptides were analysed by LC–MS/MS. The peptide sequence coverage was 69–81% (technical and biological replicates). We identified several phosphopeptides in the LC–MS analysis and the sites of phosphorylation were identified as listed in Figure 3B. We observed that abundance of phosphopeptides containing Ser^336^ was over 10-fold higher in cells with WT PCTAIRE-1 overexpression compared with those from cyclin Y alone or with KI (D304A) PCTAIRE-1 overexpression (Supplementary Figure S1), which is consistent with the results described above (Figures 2C and 2D) that Ser^336^ phosphorylation is increased in a PCTAIRE-1-dependent mechanism. This pattern was not observed for any of the remaining phosphopeptides (Supplementary Figure S1).

### Mutagenesis analysis revealed two key phosphorylation sites on cyclin Y required for binding and activating PCTAIRE-1

**Figure 4:**
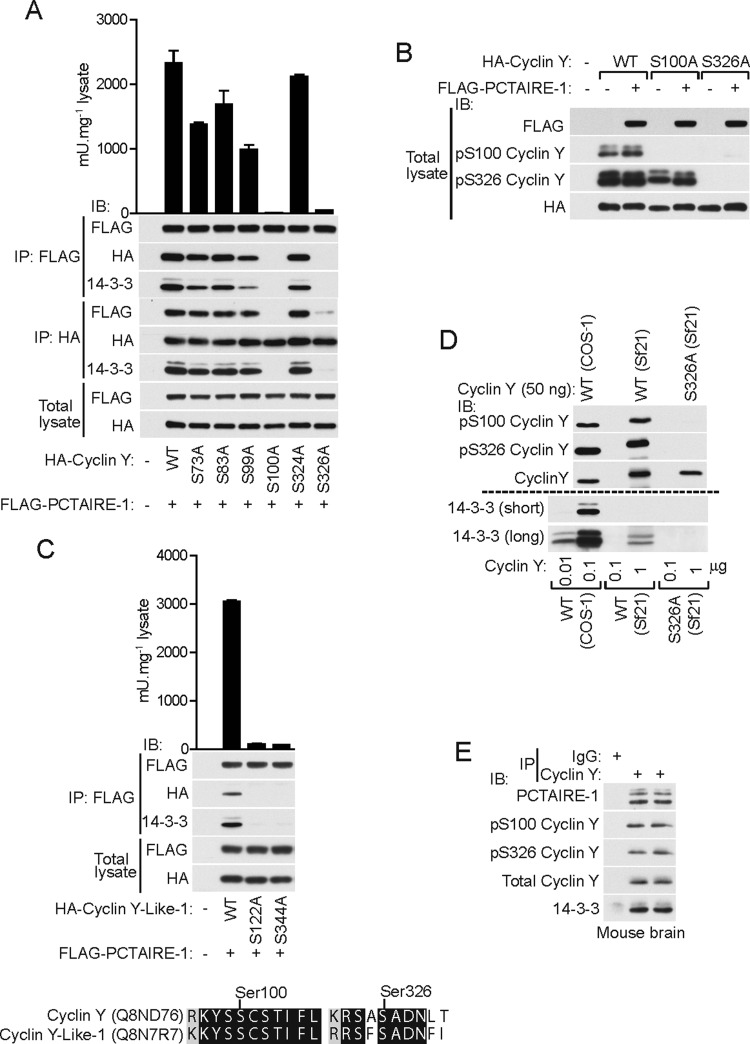
Phosphorylation of Ser^100^ and Ser^326^ on cyclin Y are essential for binding PCTAIRE-1 and 14-3-3 (**A**) Serine-to-alanine phospho-mutants of cyclin Y sites identified by MS (Figure 3) were co-expressed with FLAG–WT PCTAIRE-1 in COS-1 cells and total lysates were immunoblotted (IB) using the indicated antibodies. Additionally, lysates were immunoprecipitated (IP) using either FLAG–or HA–agarose and either immunoblotted or the FLAG-immunoprecipitates assayed for PCTAIRE-1 kinase activity. (**B**) HA–WT cyclin Y or S100A and S326A mutants were expressed in COS-1 cells with or without FLAG–WT PCTAIRE-1 and lysates were immunoblotted using the indicated antibodies. (**C**) HA–WT cyclin Y-like-1 or S122A and S344A mutants (corresponding to Ser^100^ and Ser^326^ in human cyclin Y respectively), were co-expressed with FLAG–WT PCTAIRE-1 in COS-1 cells and total lysates were immunoblotted using the indicated antibodies. Lysates were further immunoprecipitated using FLAG–agarose and either immunoblotted or assayed for PCTAIRE-1 kinase activity. Shown below is a global pairwise alignment of human cyclin Y and cyclin Y-like-1 showing amino acid sequences surrounding Ser^100^ and Ser^326^. (**D**) Immunoblot using the indicated antibodies of defined amounts of purified human cyclin Y (WT or S326A) expressed and purified either in COS-1 cells (FLAG-tagged) or Sf21 cells (His_6_-tagged). Results are expressed as means±S.D. and are representative of three independent experiments. (**E**) Mouse brain lysates (0.1–1 mg) were immunoprecipitated using anti-cyclin Y antibody and the immunoprecipitates were immunoblotted with the indicated antibodies. Results are representative of three independent experiments (*n*=4).

We next sought to determine whether any of the newly identified phosphorylation sites have key role(s) for interaction and activation of PCTAIRE-1. For this purpose, we generated a series of phospho-deficient mutants of human HA–cyclin Y and co-transfected each mutant together with FLAG–WT PCTAIRE-1 in COS-1 cells. Strikingly, we found that two point mutants, S100A and S326A, were totally incapable of binding and activating PCTAIRE-1, whereas other mutants show either full or partial binding/activation capacity (Figure 4A). Protein sequence alignment revealed that Ser^100^ and Ser^326^ are well conserved across species (Figure 3C). Phosphorylation motif analysis of human cyclin Y by Scansite [18] (http://scansite.mit.edu/) revealed a relatively high probability that these sites mediate 14-3-3 binding. Therefore, we immunoblotted anti-FLAG (PCTAIRE-1) immunoprecipitates with a anti-pan-14-3-3 antibody and observed that there was an association between the amount of cyclin Y bound to PCTAIRE-1 and 14-3-3 content in the FLAG immunoprecipitates (Figure 4A). To confirm whether Ser^100^ and Ser^326^ sites are phosphorylated in intact cells by an alternative method (i.e. immunoblot with phosphorylation site-specific antibodies) and if so, whether their levels are modulated by the presence of PCTAIRE-1, we transfected WT or mutant cyclin Y (S100A and S326A) with or without co-expression of WT PCTAIRE-1. We observed that both Ser^100^ and Ser^326^ in WT cyclin Y are phosphorylated to a similar extent in the presence or absence of PCTAIRE-1 co-expression, as judged using phospho-site-specific antibodies (Figure 4B). Interestingly, we observed that phosphorylation of Ser^100^ was abolished in the S326A mutant, whereas Ser^326^ phosphorylation was only modestly reduced in the S100A mutant. This indicates that phosphorylation of Ser^326^ may be a prerequisite for Ser^100^ phosphorylation. Given that both Ser^100^ and Ser^326^ are conserved between human cyclin Y and cyclin Y-like-1 proteins (Figure 4C), we examined whether cyclin Y-like-1 activates PCTAIRE-1 through binding that requires phosphorylation of Ser^122^ and Ser^344^ (equivalent to Ser^100^ and Ser^326^ of human cyclin Y respectively). We found that cyclin Y-like-1 bound and activated PCTAIRE-1, which was abolished in the S122A or S344A mutant (Figure 4C). We also found that 14-3-3 was only identified in PCTAIRE-1 complexed with WT cyclin Y-like-1, but not the S122A or S344A mutant. To further examine phosphorylation-dependent binding of 14-3-3 to cyclin Y, we expressed WT or S326A human His_6_–cyclin Y in insect Sf21 cells and affinity-purified recombinant cyclin Y proteins (Figure 4D). We detected similar levels of Ser^100^ and Ser^326^ phosphorylation of His_6_–WT cyclin Y compared with COS-1-derived HA–WT cyclin Y, whereas phosphorylation of Ser^100^/Ser^326^ was not detectable in the His_6_–S326A cyclin Y mutant. Immunoblotting of the purified His_6_-cyclin Y detected 14-3-3 proteins in WT, but not in the S326A mutant, even though the amount was much lower (≈100-fold less) than that detected from COS-1 derived HA–WT cyclin Y (Figure 4D). Finally, we immunoprecipitated endogenous cyclin Y from mouse brain lysates and detected phosphorylation of Ser^100^ and Ser^326^, as well as 14-3-3 and PCTAIRE-1 as binding proteins (Figure 4E). To further explore the molecular mechanism underlining the interaction between PCTAIRE-1 and cyclin Y, we generated a series of truncation and alanine mutants of cyclin Y and co-expressed each mutant with WT PCTAIRE-1 in COS-1 cells (Supplementary Figure S2A). We observed that truncation mutants lacking up to 40 N-terminal residues were still capable of binding to PCTAIRE-1 and also 14-3-3. Interestingly, however, mutants lacking the N-terminal 45 residues could no longer bind to PCTAIRE-1 or to 14-3-3. To identify the key binding region/residue(s), we generated additional mutants, particularly focusing on residues ranging from 41 to 45 of cyclin Y. We found that Δ41–45 cyclin Y mutant lost binding to both PCTAIRE-1 and 14-3-3, and alanine scanning revealed that point mutants H43A and I44A abolished binding to PCTAIRE-1 and 14-3-3 (Supplementary Figure S2B). Although this indicates that residues of the 41–45 region (particularly His^43^ and Ile^44^) play a key role in the binding of cyclin Y to PCTAIRE-1 that involves 14-3-3 binding, we cannot rule out the possibility that truncation or mutation (i.e. alanine substitution) may have disrupted normal protein folding of cyclin Y, although 14-3-3 binding is retained (although to a lower extent). To clarify this, it will be necessary to obtain structural insights (e.g. crystal structure of PCTAIRE-1–cyclin Y–14-3-3 complex).

### Activation of bacterially expressed PCTAIRE-1 requires binding of cyclin Y in association with 14-3-3

**Figure 5:**
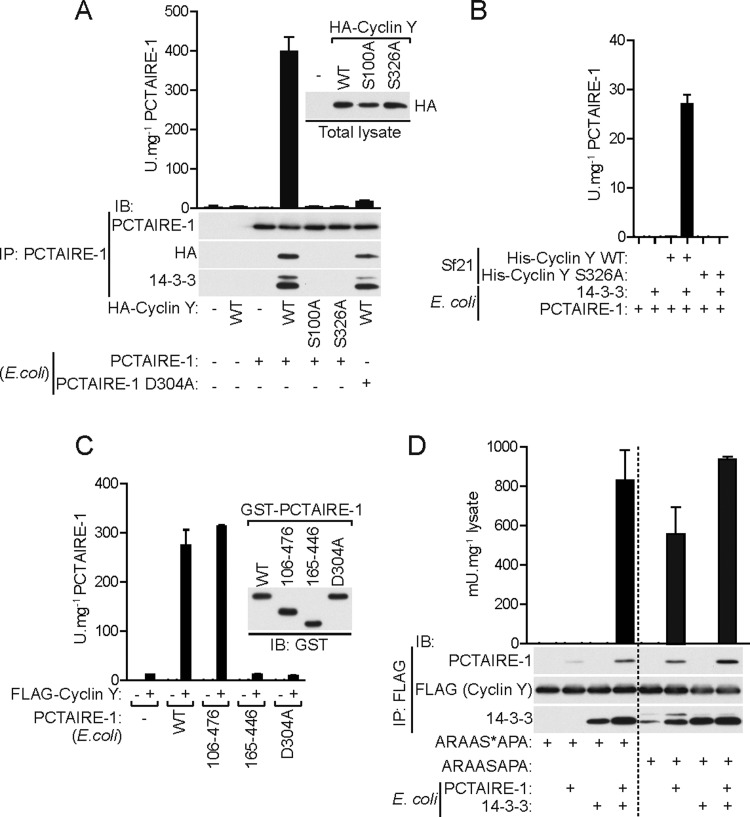
Activation of bacterially expressed PCTAIRE-1 by mammalian cyclin Y in complex with 14-3-3 (**A**) PCTAIRE-1 (100 ng), expressed and purified from *E. coli*, was incubated with COS-1 lysates (200 μg) ectopically expressing the indicated HA–cyclin Y constructs and immunoprecipitated (IP) using anti-PCTAIRE-1 (G6.1) antibody. Then, 30% of the immunoprecipitated material was immunoblotted (IB) using the indicated antibodies and 10% was assayed for kinase activity. (**B**) *E. coli*-purified PCTAIRE-1 (1 μg) was incubated with 0.5 μg of Sf21-purified His–WT cyclin Y or S326A mutant and with 1 μg of *E. coli*-purified 14-3-3ζ, together or in isolation and assayed for kinase activity. (**C**) A 100 ng amount of each of four *E. coli*-purified GST–PCTAIRE-1 proteins [WT (full-length), 106–476 truncation, kinase-domain (165–446) and KI (D304A) mutant] were incubated with 0.25 μg of COS-1-purified FLAG–cyclin Y and assayed for kinase activity. Immunoblotting was performed using GST-tagged PCTAIRE-1 proteins and activity assay was performed using GST-cleaved PCTAIRE-1 proteins. (**D**) FLAG–cyclin Y was immunoprecipitated from 50 μg of transfected COS-1 lysates using FLAG–agarose and incubated with 1 mM ARAAS*APA/14-3-3-binding phosphopeptide (* indicates phosphoserine) for 20 min at room temperature. Non-phosphopeptide ARAASAPA was used as a negative control. After washing, 100 ng of PCTAIRE-1 and 0.5 μg of 14-3-3ζ (both expressed and purified from *E. coli*), were then added together or in isolation and incubated with the immunoprecipitated protein for a further 20 min at room temperature. Samples were washed and 30% was immunoblotted using the indicated antibodies and 20% was assayed for PCTAIRE-1 kinase activity. Results are expressed as means± S.D. and are representative of three independent experiments.

It has been reported that bacterially expressed PCTAIRE-1 (bPCTAIRE-1) is catalytically inactive [3]. In addition, we have observed that co-incubation with bacterially expressed cyclin Y does not activate bPCTAIRE-1 in a cell-free assay (results not shown). This is unsurprising considering that our results suggest an absolute requirement for cyclin Y phosphorylation (and potentially 14-3-3 binding) for efficient binding/activation of PCTAIRE-1. This suggests that bPCTAIRE-1 may be activated by the cyclin Y–14-3-3 complex isolated from eukaryotic cells. We therefore incubated bPCTAIRE-1 with or without COS-1 lysates ectopically expressing WT or phospho-deficient/14-3-3-binding-deficient mutants (S100A or S326A) of cyclin Y, immunoprecipitated using anti-PCTAIRE-1 antibody and assessed binding and kinase activity (Figure 5A). As expected, bPCTAIRE-1 on its own was inactive. In contrast, WT cyclin Y (in complex with 14-3-3), but not S100A or S326A, bound and robustly activated bPCTAIRE-1. To rule out the possibility that the observed activity was due to the presence of a contaminating kinase in the purified HA–WT cyclin Y preparation, we also incubated the WT cyclin Y with KI (D304A) bPCTAIRE-1. This confirmed that the observed activity is intrinsic to bPCTAIRE-1. We also performed a similar experiment using Sf21-derived cyclin Y preparations (Figure 4D) and again observed that WT, but not S326A, activated bPCTAIRE-1 but only in the presence of additional recombinant 14-3-3 (of low abundance in Sf21-derived cyclin Y) suggesting that the formation of a ternary complex of PCTAIRE-1–cyclin-Y–14-3-3 is essential for kinase activity (Figure 5B). In line with a previous report (using a preparation of PCTAIRE-1 from mammalian cells) [5], partially truncated forms (Δ1–105 and Δ477–496/106–476), but not the kinase domain fragment (165–446) of bPCTAIRE-1, could be activated when co-incubated with COS-1 cell-derived WT cyclin Y preparation (Figure 5C), indicating the importance/requirement of regions N- and C-terminal to/flanking the kinase domain for cyclin Y-mediated PCTAIRE-1 activation. To further demonstrate that 14-3-3 is necessary for cyclin Y binding and activation of bPCTAIRE-1, we measured cyclin Y binding and activation of bPCTAIRE-1 after addition of the 14-3-3-binding phosphopeptide ARAAS*APA (where S* denotes phosphoserine). Non-phosphopeptide (ARAASAPA) was used as a negative control (Figure 5D). FLAG–WT cyclin Y was immunoprecipitated from COS-1 cells followed by incubation with the 14-3-3-binding phosphopeptide/ARAAS*APA. After washing of unbound/dissociated proteins, along with excess phosphopeptide, bPCTAIRE-1 and 14-3-3 were added/incubated together or in isolation with the immunoprecipitate, washed and then analysed by immunoblotting and kinase activity assay. We observed that the 14-3-3-binding phosphopeptide/ARAAS*APA severely diminished cyclin Y interaction with bPCTAIRE-1 and abolished bPCTAIRE-1 activity, which was restored when 14-3-3 proteins were exogenously added. In contrast, control peptide (non-phosphopeptide/ARAASAPA) did not affect cyclin Y binding to bPCTAIRE-1 or the consequent bPCTAIRE-1 activation.

### Likely disease-associated variants of CDK16/PCTAIRE-1 cause loss-of function

**Figure 6:**
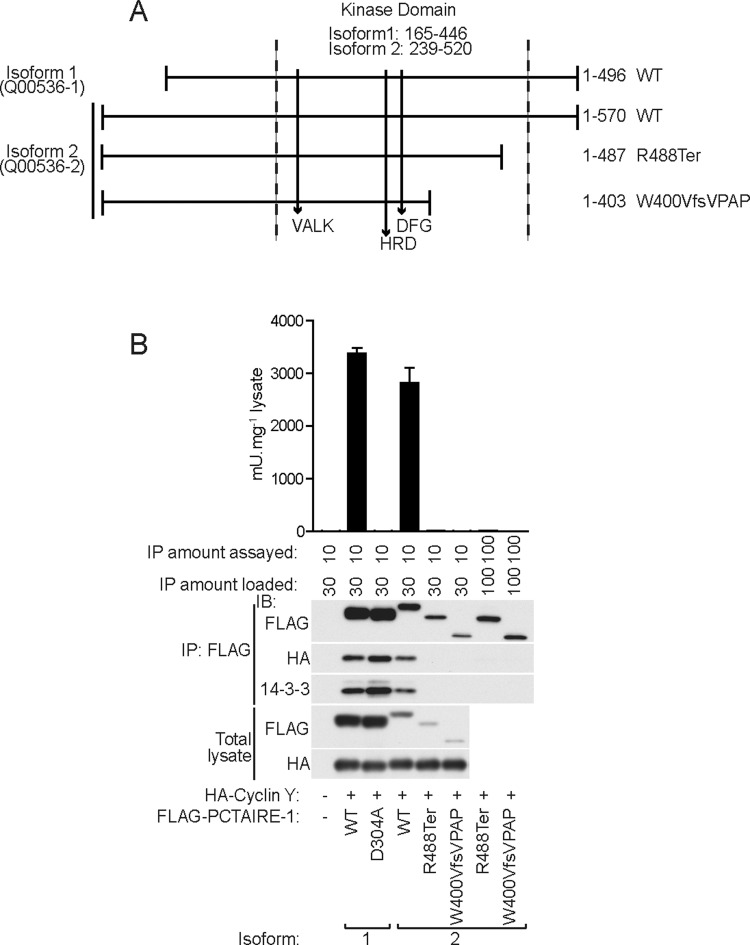
Disease-associated human PCTAIRE-1 variants abolish cyclin Y binding and kinase activity (**A**) Diagram showing relative start and end positions of human PCTAIRE-1 isoform 1 and 2 (Uni Prot accession numbers in parenthese) with respect to the kinase domain (area between vertical dashed lines). Relative positions of motifs required for an active kinase (VALK, HRD and DFG motifs) are indicated by vertical unbroken lines pointing downwards. Disease-associated variants in isoform 2 that lead to truncations are shown on the right. For both isoforms, amino acid residues of the kinase domain and total length are indicated by numbers. (**B**) Isoform 1 of FLAG–WT PCTAIRE-1 or KI (D304A) mutant, and isoform 2 of FLAG–WT PCTAIRE-1 or disease-associated variants (R488Ter and W400VfsVPAP) were co-expressed with HA–WT cyclin Y in COS-1 cells and total lysates were immunoblotted (IB) using the indicated antibodies. Lysates were immunoprecipitated (IP) using FLAG–agarose and either assayed for kinase activity or immunoblotted using the indicated antibodies. Additional increased amounts of disease-associated variants assayed or immunoblotted after immunoprecipitation are also indicated. Results are expressed as mean±S.D. and are representative of three independent experiments.

X-linked intellectual disability (XLID) is a clinically and genetically heterogeneous disorder with >100 genes identified so far. For some of these XLID genes, only very few families with pathogenic variants have so far been reported, suggesting that mutations in these genes are very rare. In our recent study of 405 families with XLID investigated by X chromosome exome sequencing, we identified a variant in *CDK16/PCTAIRE-1* in a family with four affected males who all carry the variant and their mothers are heterozygous carriers [chrX:47086041-47086043, delTG, Q00536-2 (isoform 2): p.Trp400ValfsVPAP]. This result led us to propose that *CDK16*/*PCTAIRE-1* is a novel candidate XLID gene [19]. An additional protein truncating *CDK16*/*PCTAIRE-1* variant, classified as pathogenic or probably pathogenic (probably affecting function) has been identified in an unrelated clinical case [chrX:47086497C>T, Q00536-2 (isoform 2): p.Arg488Ter] (http://genetics.emory.edu/egl/emvclass/emvclass.php and ClinVar database (http://www.ncbi.nlm.nih.gov/clinvar/variation/166819/). Thus we investigated whether these variants could have an effect on cyclin Y–14-3-3 binding and also on PCTAIRE-1 enzymatic activity. For this purpose, we ectopically expressed both variants of human PCTAIRE-1 (FLAG–PCTAIRE-1 Trp400ValfsVPAP and FLAG–PCTAIRE-1 Arg488Ter) and its reference WT (isoform 2) together with WT cyclin Y (Figures 6A and 6B). Isoform 2 has a 74 amino acid extension at its N-terminus compared with isoform 1 [which has been used throughout the present study (except studies shown in Figure 6) and by others]. PCTAIRE-1 isoform 2 showed modestly lower expression compared with isoform 1, but displayed relatively comparable binding to cyclin Y–14-3-3 as well as activity to that of isoform 1. We observed both pathogenic variants consistently expressed much lower than their reference WT isoform 2 (Figure 6B), suggesting that they may be less stable than WT PCTAIRE-1 isoform 1. The variants were catalytically inactive {although they still contain key features of a functional kinase such as the VALK, HRD and HFD motifs [20] (Figure 6A)} and they displayed no or marginally detectable binding to cyclin Y, even when the amount of loading was matched to that of WT (isoform 2) (Figure 6B; Supplementary Figure S3).

## DISCUSSION

Several studies, including our own, have recently established that activation of PCTAIRE-1/CDK16 requires binding of cyclin Y [5,6,13,16]; however, the underlying molecular mechanism that mediates this interaction was elusive. We demonstrate in the present study that phosphorylation of cyclin Y (Ser^100^ and/or Ser^326^) and also cyclin Y-like-1 protein (Ser^122^ and/or Ser^344^, equivalent to Ser^100^ and Ser^326^ of cyclin Y respectively) promotes binding to 14-3-3 proteins in which both events are necessary for binding to, and activation of, PCTAIRE-1.

Given that residues surrounding Ser^12^ and Ser^336^ comprise the preferred consensus sequence for PCTAIRE-1, our initial hypothesis was that PCTAIRE-1 phosphorylates cyclin Y and that this modification influences their interaction. We identified Ser^336^, but not Ser^12^, as a PCTAIRE-1-dependent phosphorylation site; however, contrary to our hypothesis, phospho-deficient S336A mutant displayed normal binding to PCTAIRE-1. Although the functional role of Ser^336^ phosphorylation is still unknown, monitoring the phosphorylation of Ser^336^ may be a useful read-out to assess activity of PCTAIRE-1 in intact cells/tissues in the absence of well-characterized downstream substrates.

To our knowledge, the present study is the first study demonstrating that the interaction between PCTAIRE-1 and cyclin Y requires phosphorylation of cyclin Y that results in 14-3-3 binding. However, it should be noted that a recent study by Li et al. [21] has reported cyclin Y binding of 14-3-3, which was associated with interaction with a closely related kinase of PCTAIRE-1, namely PFTAIRE-1/CDK14. They showed that the S100A/S326A double mutant of cyclin Y failed to interact with 14-3-3 and PFTAIRE-1/CDK14. However, Li et al. did not provide evidence that Ser^100^ and Ser^326^ were indeed phosphorylated in cells. A follow-up study by the same group claimed that they identified several phosphorylation sites on cyclin Y using MS; however, they did not provide data regarding the identity of specific phosphopeptides [22]. Surrounding sequences of the identified phosphopeptides (that include Ser^100^ or Ser^326^) in the present study contain multiple serine residues (Figure 3B). The phosphorylated residues were confidently assigned from the MS/MS spectra using bioinformatics tools, but we further validated the phosphorylation of both sites using phospho-site-specific antibodies. Although we observed that a single serine substitution by a non-phosphorylatable alanine residue, S100A or S326A, was sufficient to abolish 14-3-3 binding, the S326A mutation resulted in ablation of Ser^100^ phosphorylation. This indicates that phosphorylation of Ser^326^ may be a prerequisite event for phosphorylation of Ser^100^ or alternatively the S326A mutation prevented the upstream kinase(s) from phosphorylating the Ser^100^ residue. It is unclear therefore whether Ser^100^ phosphorylation alone is sufficient for 14-3-3 binding or whether Ser^326^ phosphorylation also plays a role.

In cultured cell lines, PCTAIRE-1 is phosphorylated at several residues, including Ser^119^ and Ser^153^. These sites have been shown to be substrates for protein kinase A (PKA) *in vitro* [3] and also in intact cells [5] in the case of Ser^153^. Phosphorylation of Ser^119^ and Ser^153^ creates 14-3-3 consensus binding motifs, whose binding activity has been confirmed by overexpression and mutagenesis study [3]. However, the function of this modification and 14-3-3 binding has not been resolved. Given that we were able to activate bPCTAIRE-1 by co-incubating with COS-1-derived/purified cyclin Y in complex with 14-3-3, it is unlikely that direct binding of 14-3-3 to PCTAIRE-1 is an essential mechanism by which PCTAIRE-1 is activated.

As part of systematic X-chromosome exome re-sequencing of index patients from 405 families with XLID, a unique CDK16/PCTAIRE-1 variant was identified in a multigenerational family [19]. In addition, evidence from other studies suggests a role for PCTAIRE-1 in brain function. In more detail, in extreme fatal primary human microcephaly, due to a deleterious variant in megakaryoblastic leukemia 2 (MKL2) [23] and in MKL1/MKL2 double knockout mice with defective neuronal migration and neurite outgrowth during brain development, PCTAIRE-1 expression is decreased [7]. Also, targeted overexpression of PCTAIRE-1 into rat hippocampus with virus-mediated gene transfer caused memory deficits [24]. Combined with the results of this study showing that patient-related truncated protein mutants are unstable and catalytically inactive and therefore loss of protein function mutations, these data suggest that PCTAIRE-1 plays an important role for normal brain development and function and hence its expression and activity need to be tightly regulated.

In summary, our work has provided a key mechanistic insight into the regulation/activation of PCTAIRE-1, which is mediated through binding of phosphorylated cyclin Y in complex with 14-3-3 protein. CDK16/PCTAIRE-1 has emerged as a potential key player for normal brain development and function, but further studies are required to identify downstream molecules/pathways that link PCTAIRE-1 and biological/physiological processes in brain.

## Abbreviations

bPCTAIRE-1: bacterially expressed PCTAIRE-1
CDK: cyclin-dependent kinase
Eip63E: ecdysone-induced protein encoded by a gene at chromosomal position 63E
HA: haemagglutinin
HRP: horseradish peroxidase
KI: kinase-inactive
MKL: megakaryoblastic leukemia
TBST: tris buffered saline with Tween-20
TEAB: triethylammonium bicarbonate
WT: wild-type
XLID: X-linked intellectual disability
